# Energy expenditure of rugby players during a 14-day in-season period, measured using doubly labelled water

**DOI:** 10.1007/s00421-018-3804-4

**Published:** 2018-01-20

**Authors:** Deborah R. Smith, R. F. G. J. King, L. C. Duckworth, L. Sutton, T. Preston, J. P. O’Hara, B. Jones

**Affiliations:** 1 Institute for Sport, Physical Activity and Leisure, Leeds Beckett University, G19 Fairfax Hall, Headingley Campus, Leeds, LS6 3QN UK; 2 Stable Isotope Biochemistry Laboratory, Scottish Universities Environmental Research Centre, East Kilbride, UK; 3 Leeds Rhinos RLFC, Leeds, UK; 4 Yorkshire Carnegie RUFC, Leeds, UK; 5 The Rugby Football League, Leeds, UK

**Keywords:** Team sport, Rugby, Adolescent, Athletes, Metabolic rate, Energy requirements, Energy expenditure, Doubly labelled water, Physical activity level, Nutrition

## Abstract

Criterion data for total energy expenditure (TEE) in elite rugby are lacking, which prediction equations may not reflect accurately. This study quantified TEE of 27 elite male rugby league (RL) and rugby union (RU) players (U16, U20, U24 age groups) during a 14-day in-season period using doubly labelled water (DLW). Measured TEE was also compared to estimated, using prediction equations. Resting metabolic rate (RMR) was measured using indirect calorimetry, and physical activity level (PAL) estimated (TEE:RMR). Differences in measured TEE were unclear by code and age (RL 4369 ± 979; RU 4365 ± 1122; U16, 4010 ± 744; U20, 4414 ± 688; U24, 4761 ± 1523 Kcal day^− 1^). Differences in PAL (overall mean 2.0 ± 0.4) were unclear. Very likely differences were observed in RMR by code (RL 2366 ± 296; RU 2123 ± 269 Kcal day^− 1^). Differences in relative RMR between U20 and U24 were very likely (U16, 27 ± 4; U20, 23 ± 3; U24, 26 ± 5 Kcal kg^− 1^ day^− 1^). Differences were observed between measured and estimated TEE, using Schofield, Cunningham and Harris–Benedict equations for U16 (187 ± 614, unclear; − 489 ± 564, likely and − 90 ± 579, unclear Kcal day^− 1^), U20 (− 449 ± 698, likely; − 785 ± 650, very likely and − 452 ± 684, likely Kcal day^− 1^) and U24 players (− 428 ± 1292; − 605 ± 1493 and − 461 ± 1314 Kcal day^− 1^, all unclear). Rugby players have high TEE, which should be acknowledged. Large inter-player variability in TEE was observed demonstrating heterogeneity within groups, thus published equations may not appropriately estimate TEE.

## Introduction

Rugby league (RL) and rugby union (RU) are high-intensity intermittent team sports, with the addition of tackles and collisions (Johnston et al. 2014; Read et al. 2017). During match play RL players typically cover a greater distance than RU players (Duthie et al. 2003; Twist et al. 2014), whereas RU players are usually involved in a greater number of static exertions (Cahill et al. 2013; Twist et al. 2014). During match play, 47 ± 12 collisions have been observed in RL players (Johnston et al. 2014) and 91 ± 19 static exertions in RU players (Roberts et al. 2008). Tackle and collision-based activities within both codes of rugby, which occur during match play and training, have a high energy cost (Highton et al. 2017). As such, it is likely that rugby is associated with distinct energy expenditures compared to other non-contact team sports, such as soccer and basketball. However, criterion data of energy expenditure are lacking in rugby players.

Energy expenditure for senior RU players has been estimated using SenseWare armbands during an in-season period, where forwards and backs expended 3800 ± 120 and 3346 ± 120 Kcal day^− 1^, respectively (Bradley et al. 2015). However, total energy expenditure (TEE) may have been under-estimated, as the armbands could not be worn during water based or contact activities. An alternative method of assessing energy expenditure is doubly labelled water (DLW). This non-invasive gold standard method accounts for all daily activities in free-living individuals (Schoeller 1999; Westerterp 2017). In-season energy expenditure measured using DLW has only been previously reported in senior RL players (Morehen et al. 2016), with values reported to be 5,378 ± 645 Kcal day^− 1^, far greater than that previously reported in senior RU players (Bradley et al. 2015). While the energy expenditure of senior RL and RU players have been quantified using various methods, no study to date has reported the energy demands of academy rugby players.

Younger rugby players may have unique energy requirements, given the fact they are still growing and developing physically (Desbrow et al. 2014; Jones et al. 2017). Energy demands for 15–18 year olds are higher than at any other life-stage (COMA 1991). However, previous research in academy rugby players has demonstrated that some age groups may under-achieve energy demands for growth, maturation and daily activity (Smith et al. 2016). Therefore, it is challenging to define the energy requirements of younger athletes, especially in a sport where the energy expenditure is unknown.

Energy requirements are often estimated using basal metabolic rate (BMR) (Schofield 1984) or resting metabolic rate (RMR) equations that take into account sex, age and body mass (Roza and Shizgal 1984), or fat free mass (FFM) (Cunningham 1991). Although the Schofield equation has been recommended as an estimate of BMR in adolescents (Desbrow et al. 2014), it has recently been suggested that the Cunningham and Harris–Benedict equations may provide a more reasonable estimate of RMR in athletes (ACSM 2016; Jagim et al. 2017). Although the latter were developed in healthy populations, these were non-athletic individuals and the age range did not include adolescents. The Schofield equation was also developed using non-athletic individuals, but included younger age groups. Consequently, these equations are not specific to individuals that frequently train and compete, thus have potential to over- or under-estimate metabolic rate. This may be due to differences in lean body mass (Jagim et al. 2017), which could be emphasised further for rugby players that have a greater body mass than other athletic populations (Santos et al. 2014).

Metabolic rate is often multiplied by a physical activity level (PAL) to predict TEE, which has been reported as 2.9 for RL players during an in-season period (Morehen et al. 2016). TEE consists of energy from metabolic rate, the thermic effect of feeding (TEF) and thermogenesis from activity, where the latter comprises of both exercise and non-exercise activity (ACSM 2016; Speakman and Selman 2003). Previous studies have predicted TEE using a PAL of 2.0 (Lundy et al. 2006) and 1.86 (Tooley et al. 2015), where estimated energy requirements were thought to be under-achieved. However, Tooley et al. (2015) also accounted for the TEF which means that the PAL was adjusted. The energy demands for 15–18 year olds are higher than at any other life-stage (COMA 1991; Desbrow et al. 2014), thus growth and maturation of adolescent athletes may also increase TEE. Since sport and age specific nutrition recommendations are lacking, it is important to use criterion measures that account for all components of TEE, to better inform practise.

The purpose of this study was to quantify the mean daily energy expenditure of elite rugby players by code (i.e., RL and RU) and age (i.e., U16 vs. U20 vs. U24) over a 14-day in-season period, using the DLW method. A second aim was to compare measured to estimated TEE, using prediction equations within the different age groups.

## Methods

### Study design

A cross-sectional design was used to investigate the energy expenditure of 27 elite male English rugby players, over 14-days. Data collection started approximately 6 months into the competitive season for both RL (June–July) and RU (March–April) players, where training exposure and match play was matched as best as possible (Table 1). Players were grouped by code (RL and RU) and age (U16, U20 and U24). All measurements explained below were undertaken on the day of the DLW bolus dose (day 0).

**Table 1:** Characteristics of elite English rugby players, including typical training exposure during the 14-day in-season period

	Rugby union			Rugby league		
	U16 (*n* = 5)	U20 (*n* = 4)	U24 (*n* = 4)	U16 (*n* = 5)	U20 (*n* = 5)	U24 (*n* = 4)
Age (years)	15.6 ± 0.5	18.3 ± 0.5	23.0 ± 0.8	15.2 ± 0.8	17.6 ± 1.1	23.0 ± 1.8
Height (cm)	182.1 ± 7.5	178.1 ± 3.5	184.4 ± 3.2	180.8 ± 7.0	176.8 ± 3.8	184.7 ± 2.5
Body mass (kg)	85.4 ± 17.3	85.1 ± 8.3	99.4 ± 16.8	79.3 ± 17.1	87.6 ± 8.8	98.3 ± 4.8
Fat free mass (kg)	67.8 ± 5.0	68.9 ± 7.4	77.5 ± 7.5	62.2 ± 10.6	66.4 ± 7.3	82.1 ± 4.8
Light training days^a^	0–2	1–2	1–3	4	2	8–9
Heavy training days^b^	3–5	4–7	4	3	7	1
Rugby match	0–2	0–2	1	0–2	1	0–2
Rest day^c^	9–10	6–9	7–9	8	5	4

^a^Light day consisted of one resistance or one rugby training session

^b^Heavy day consisted of both resistance and rugby training sessions

^c^Rest days were when no training with the club was scheduled

### Participants

Characteristics of players and training exposure over the recording period are shown in Table 1. Players were recruited from a professional Super League RL club and their respective academy, and a professional Championship RU club and their respective Regional Academy. All academy (i.e., RL & RU) and RU senior players were classified as successful elite athletes, and RL senior players were world class elite (Swann et al. 2015). Ethics approval for the study was granted by Carnegie Faculty Research Ethics Committee, Leeds Beckett University. This was following approval of the proposed ionising radiation exposure to both participants and operators, by a qualified medical physics expert and radiation protection advisor. Informed consent was obtained from all individual participants included in the study. Additionally, where appropriate, informed player assent and parental consent was obtained (for under 18-year-olds).

### Anthropometrics and body composition

Players were measured wearing lightweight clothing with jewellery removed. Height and body mass was measured using a stadiometer (Seca Alpha, Birmingham, UK) and calibrated scales (Seca Alpha 220, Birmingham, UK) to the nearest 0.1 cm and 0.1 kg, respectively. Dual X-ray absorptiometry scans (DXA) were conducted on a fan-beam GE Lunar iDXA using standard and thick mode where players body thickness was > 25 cm. A trained technologist conducted and analysed all DXA scans following National Health and Nutrition Examination Survey recommendations as advised by the International Society for Clinical Densitometry guidelines for patient positioning (Hangartner et al. 2013). The primary outcome used from the DXA scan for this study was FFM, which was used for all relative calculations and where required in prediction equations.

### Resting metabolic rate

Following the protocol of Matarese (1997) indirect calorimetry was used to measure oxygen (O_2_) consumption and carbon dioxide (CO_2_) production. Players reported to the laboratory following an overnight fast and were asked to rest, but not sleep, in a supine position for 30 min prior to measurement of RMR. Wearing a sterile mask, attached to a metabolic cart (*V*_max_, Encore 29, USA), players were asked to breathe normally until a steady state was reached. This was determined after ~ 15 min, where a steady state was defined as a single 5-min interval, where the average minute O_2_ consumption and CO_2_ changed by less than 10%, and the average respiratory quotient changed by less than 5%. RMR was computed using the Weir (1949) equation. Data for one RL U16 player was omitted as he was uncomfortable completing this aspect of data collection.

### Daily energy expenditure

TEE was measured over a 14-day period. Bolus loads of ^2^H_2_^18^O (DLW) determined by the method of IAEA (2009), were administered to players during their respective in-season period. A stock mixture of ^2^H_2_O (99 atom %) and H_2_^18^O (10 atom %) was prepared and mixed, from which individual doses of 66 g were prepared gravimetrically [based on 0.12 g kg^− 1^ (99% ^2^H_2_O) and 1.8 g kg^− 1^ H_2_^18^O (10%) of the largest body mass expected in the study]. Doses were calculated to target an initial enrichment > 150 ppm in excess of ^2^H and ^18^O baseline. A dilution of the dose stock was also prepared to aid calibration of the mass spectrometer measurement.

Players collected 8 × 20 ml urine samples over the 14-day period. A baseline sample was collected immediately prior to the DLW bolus dose (day 0), and during the second void on days 1, 2, 3, 7, 12, 13 and 14. This allowed for mean values to be calculated, thus reducing analytical error. Players noted the date and time of sample collection, before handing these to the principal researcher in tightly sealed universal containers. All samples were filtered to an acellular state to comply with the Human Tissue Act and stored in cryovials at − 40 °C, before dispatching to the Stable Isotope Biochemistry Laboratory, Scottish Universities Environmental Research Centre.

Isotopic abundance was measured by continuous-flow isotope ratio using mass spectrometry following gaseous exchange, for both isotopes (Prosser and Scrimgeour 1995). Isotopic enrichments were calculated by subtraction of the pre-dose abundance in each case. ^2^H and ^18^O elimination rates were estimated from the gradient of the log transformed data and combined with total body water from the intercept of these plots, to estimate CO_2_ production rate. Schoeller’s equation was used to estimate TEE (Goran et al. 1994). The precision of isotopic enrichment was < 2 ppm for every measurement. The mean ratio of the tracer elimination rate was within normal range (1.336) and tracer enrichment remained above the minimum recommended at the end of the study, in every case (IAEA 2009).

### Estimation of energy requirements

Three prediction equations were used to estimate metabolic rate; the Schofield equation (Schofield 1984), which is recommended for prediction of BMR in the UK population (COMA 1991), the Harris–Benedict (Roza and Shizgal 1984) and Cunningham (1991) equations. The latter have been reported to predict RMR more accurately in adolescent soccer players (De Lorenzo et al. 1999), and more recently have been recommended for use with athletes (ACSM 2016). Finally, to estimate TEE, predicted metabolic rate was multiplied by the estimated PAL, derived from criterion measures (measured TEE divided by measured RMR).

### Analyses of data

Data are presented as means ± standard deviation, and analysed by code (i.e., RL vs. RU) then consecutive age (i.e., U16 vs. U20 vs. U24). Further analyses, by code and age, were undertaken for RMR and TEE. Data for prediction equations were analysed by age group only, due to some equations using different calculations based on age and the small sample sizes when grouped by code and age.

For statistical analyses all data were log transformed to reduce bias. Effect sizes were calculated and interpreted as trivial (< 0.2), small (0.2–0.6), moderate (0.6–1.2), large (1.2–2.0), very large (2.0–4.0) or extremely large (> 4.0) (Hopkins et al. 2009). Magnitude based inferences were calculated for practical significance (Hopkins et al. 2009). The threshold used for the observed change was 0.2 (mean difference divided by between subject SD) (Deighton et al. 2017). Magnitudes for the observed change, based on 90% confidence interval (CI), were most unlikely (< 0.5%); very unlikely (0.5–5%); unlikely (5–25%); possibly (25–75%); likely (75–95%); very likely (95–99.5%); or most likely (> 99.5%) (Hopkins 2007). Effects with CI crossing upper and lower boundaries of the smallest worthwhile difference (± 0.2), were described as unclear (Hopkins 2007).

## Results

Table 1 shows player characteristics. Unclear differences for body mass and FFM were observed between codes [RL vs. RU: *d* = − 0.11 (− 0.75 to 0.53) and *d* = − 0.22 (− 0.86 to 0.41), respectively]. Differences in body mass and FFM for U16 vs. U20 were unclear [*d* = − 0.37 (− 1.12 to 0.39) and *d* = − 0.33 (− 1.09 to 0.43)]. U24 players were very likely heavier than U20 players [*d* = − 1.17 (− 1.99 to − 0.35)], and most likely had a greater FFM [*d* = − 1.74 (− 2.54 to − 0.93), respectively].

### Resting metabolic rate and total energy expenditure

**Fig. 1:**
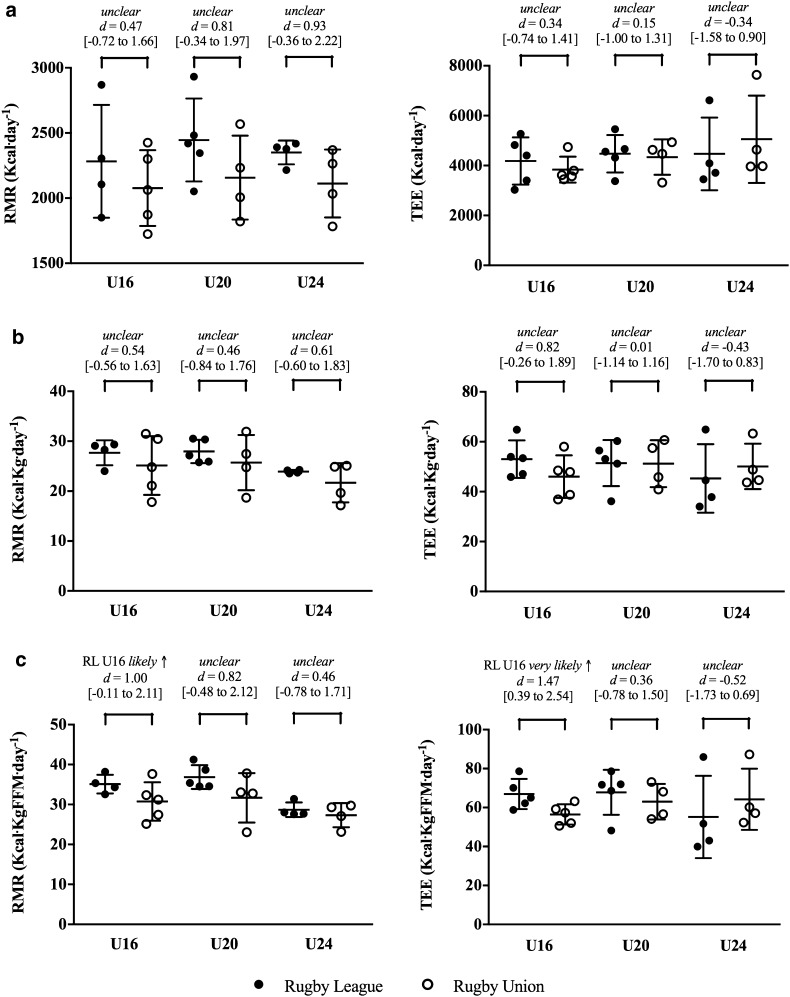
Resting metabolic rate and total energy expenditure in elite rugby players. Lines represent the mean and 90% confidence intervals

It was likely and very likely that RMR was greater for RL than RU players (Table 2). By age, RMR was unclear between consecutive age groups (Table 3). Relative to body mass, RMR and FFM were very likely greater for U20 than U24 players. Negligible differences were observed between RL and RU players for TEE (Table 2). Despite ~ 350–400 Kcal day^− 1^ differences between consecutive age groups (Table 3), differences in TEE were unclear due to large individual variation. The mean PAL was 2.0 ± 0.4 for all players and differences were unclear by code and age. Figure 1 summarises RMR and TEE for age groups by code.

**Table 2:** Resting metabolic rate and total energy expenditure of elite English rugby league and rugby union players, during an in-season period

RMR	RL (*n* = 13)	RU (*n* = 13)	RL vs. RU
Kcal day^− 1^	2366 ± 296	2123 ± 269	0.87 (0.22 to 1.53) Very likely
Kcal kg day^− 1^	29 ± 3	24 ± 5	0.63 (− 0.02 to 1.28) Likely
Kcal kg FFM day^− 1^	34 ± 4	30 ± 5	0.82 (0.17 to 1.4) Likely

TEE	RL (*n* = 14)	RU (*n* = 13)	RL vs. RU
Kcal day^− 1^	4369 ± 979	4365 ± 1122	0.01 (− 0.62 to 0.65) Unclear
Kcal kg day^− 1^	50 ± 10	49 ± 9	0.12 (− 0.52 to 0.75) Unclear
Kcal kg FFM day^− 1^	64 ± 14	61 ± 10	0.17 (− 0.46 to 0.81) Unclear

PAL	RL (n = 14)	RU (n = 13)	RL vs. RU
TEE:RMR	1.90 ± 0.36	2.07 ± 0.46	− 0.42 (− 1.07 to 0.23) Unclear

Data between groups are presented as Cohen’s *d* effect size (90% confidence intervals) and magnitude based inference

**Table 3:** Resting metabolic rate and total energy expenditure of elite English rugby players from academy to senior level, during an in-season period

RMR	U16 (*n* = 9)	U20 (*n* = 9)	U24 (*n* = 8)	U16 vs. U20	U20 vs. U24
Kcal day^− 1^	2168 ± 353	2318 ± 335	2232 ± 221	− 0.43 (− 1.22 to 0.36) Unclear	0.25 (− 0.55 to 1.05) Unclear
Kcal kg day^− 1^	26 ± 5	27 ± 4	23 ± 3	− 0.16 (− 0.95 to 0.62) Unclear	1.05 (0.24 to 1.86) Very likely
Kcal kg FFM day^− 1^	33 ± 4	35 ± 5	28 ± 2	− 0.33 (− 0.11 to 0.46) Unclear	1.42 (0.63 to 2.22) Very likely

TEE	U16 (*n* = 10)	U20 (*n* = 9)	U24 (*n* = 8)	U16 vs. U20	U20 vs. U24
Kcal day^− 1^	4010 ± 744	4414 ± 688	4761 ± 1523	− 0.55 (− 1.31 to 0.22) Unclear	− 0.19 (− 1.03 to 0.64) Unclear
Kcal kg day^− 1^	50 ± 8	51 ± 9	48 ± 11	− 0.19 (− 0.96 to 0.58) Unclear	0.38 (− 0.44 to 1.20) Unclear
Kcal kg FFM day^− 1^	62 ± 8	66 ± 10	60 ± 18	− 0.37 (− 1.14 to 0.40) Unclear	0.48 (− 0.35 to 1.31) Unclear

PAL	U16 (*n* = 10)	U20 (*n* = 9)	U24 (*n* = 8)	U16 vs. U20	U20 vs. U24
TEE:RMR	1.91 ± 0.20	1.93 ± 0.33	2.14 ± 0.64	− 0.01 (− 0.80 to 0.78) Unclear	− 0.32 (− 1.15 to 0.50) Unclear

Data between groups are presented as Cohen’s *d* effect size (90% confidence intervals) and magnitude based inference

### Measured vs. predicted energy expenditure

**Fig. 2:**
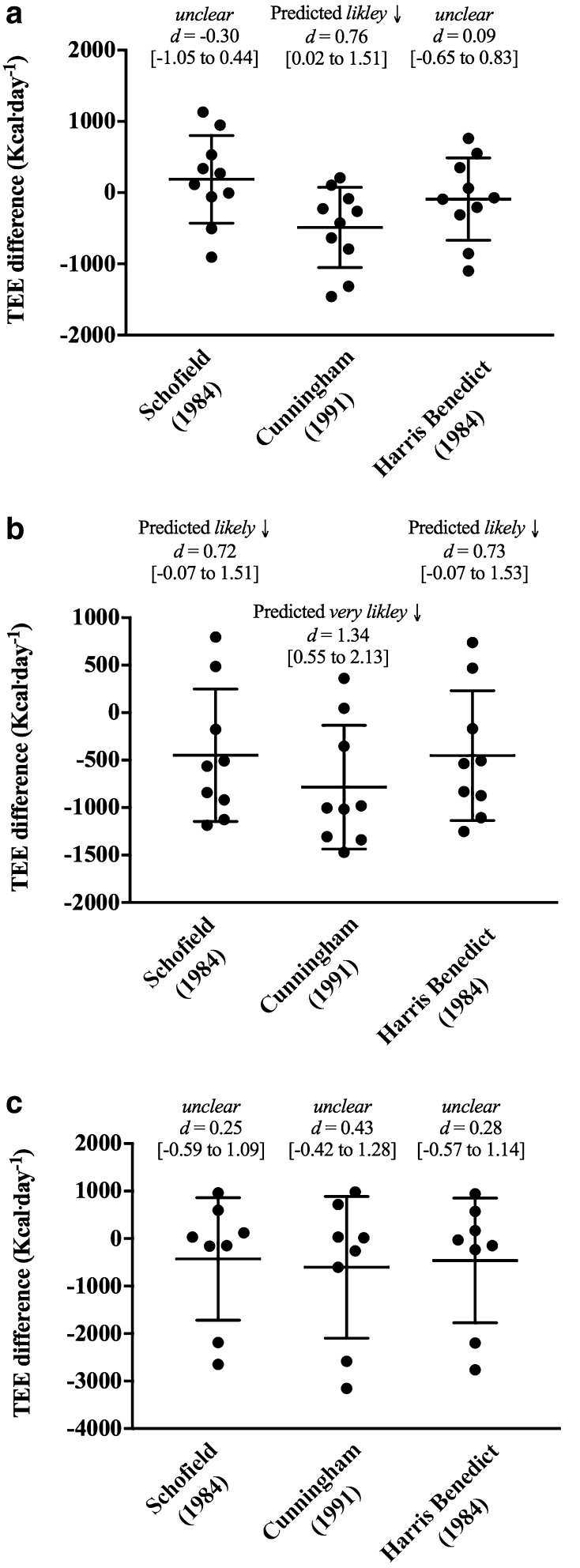
Measured and predicted total energy expenditure in elite rugby players. Lines represent the mean and 90% confidence intervals

To estimate TEE, all metabolic rate prediction equations were multiplied by the PAL of 2.0 observed in this study. When used to predict TEE, the Cunningham equation likely under-estimated for U16 players. Differences between TEE and the Schofield and Harris–Benedict equations for U16 players were unclear. The Schofield, Cunningham and Harris–Benedict equations likely, very likely and likely under-estimated TEE for U20 players, respectively (Fig. 2). Unclear differences were observed between the prediction equations and TEE for U24 players.

## Discussion

This study quantified TEE of elite adolescent and senior RL and RU players over a 14-day in-season period, using the gold standard DLW method. Despite negligible differences in TEE and PAL by code or age group, measured RMR (absolute, relative to body mass and FFM) was greater for RL than RU players, and relative RMR was lower for U24 than U20 players. Prediction equations under-estimated TEE measured by DLW in most cases, thus practitioners should consider this when supporting rugby players.

Mean TEE for RL U24 players in this study was ~ 900 Kcal day^− 1^ less than that reported for senior RL players (Morehen et al. 2016), however, the range of TEE for our cohort (3452–6617 Kcal day^− 1^) was similar to those reported by Morehen et al. (2016). Differences in the mean TEE may be due to training schedules, as players in this study had more rest days and less heavy training days. TEE for RU U24 players in the present study were ~ 500–1000 Kcal day^− 1^ greater than previously reported mean data in senior RU players (Bradley et al. 2015). The Senseware armbands, used to assess the energy demands by Bradley et al. (2015) were unable to be worn during water based or heavy contact activities, therefore, it is likely that TEE was under-estimated. The energy cost of repeated collisions has been under-estimated by 45% when using similar microtechnology, which could result in an under-estimation of up to 528 Kcal in a full 80-min rugby match (Highton et al. 2017). The differences may also represent the recovery demands (i.e., from exercise and impact-induced muscle damage) of rugby training and match play (Naughton et al. 2017), which the Senseware would be unable to quantify.

Furthermore, U24 RL and RU players in the current study expended ~ 1200 Kcal day^− 1^ more than professional soccer players of a similar age (Ebine et al. 2002). Although the training schedule of the soccer players was not presented, it was described as a moderate training week with two games, which may have been more demanding than what was observed for the U24 players in this study. As such, the greater TEE observed in rugby players may be due to the energy demands and recovery costs of a collision-based sport, in addition to differences in body mass. The mean body mass of the U24 RL and RU players in the current study was ~ 30 kg more than the soccer players (Ebine et al. 2002), which likely contributes towards the increased TEE. Consequently, it is important for practitioners working in rugby to consider the increased energy demands, in comparison to other sports that may be a result of a high body mass and the contact demands of the sport.

In-season TEE for U20 players was ~ 200 Kcal day^− 1^ less than basketball players TEE measured using DLW (Silva et al. 2013). Although the U20 players in this study has a greater body mass than basketball players (80.9 kg), our cohort had a lower FFM, trained less and played fewer games which may account for the lower TEE. Although it is challenging to make comparisons between sports, due to varying methodologies, the population specific TEE data reported in this study can be used as a reference by practitioners working with rugby players undertaking similar training and match play schedules. A limitation of the present study was that training load was not measured. Although this may not provide direct comparisons to other sports, it could be useful when making comparisons to studies that have attempted to measure TEE in rugby.

Research using the DLW method to measure TEE in U16 athletes is lacking. TEE for U16 rugby players in the current study was 375 Kcal day^− 1^ greater than previously estimated for competitive male athletes (14.8 ± 2 years) (Carlsohn et al. 2011). The U16 rugby players in this study had a greater body mass and FFM than the competitive athletes, and the methods used by Carlsohn et al. (2011) may have under-estimated TEE due to reliance on self-reported physical activity and prediction equations to measure RMR. It is important to understand the energy demands of adolescent players, to ensure energy requirements for growth and maturation are met (Smith et al. 2016). More importantly, practitioners should appreciate the range in TEE within an age group and code, and adopt individualised nutrition support, to meet the unique requirements of individual players.

Despite similar TEE between all players, a greater RMR was observed in RL players, suggesting that TEF and activity thermogenesis was less than RU players. While the number of game days were similar for RL and RU players, training schedules differed, with RL players undertaking more training. Therefore, it would be expected that energy expenditure related to activity thermogenesis was greater in RL players, but this may have been reflected in the RL players greater RMR. Alternatively, activity thermogenesis may be greater in RU due to the energy demands from the greater number of collisions observed during match play when compared to RL players (Cahill et al. 2013; Twist et al. 2014). Due to the individual variability in body mass and FFM, mean differences were unclear, and therefore it remains unknown if this contributed towards RMR.

RMR for U16 and U20 players was ~ 335 and ~ 485 Kcal day^− 1^ greater than soccer players (15.5–18.2-years-old) (De Lorenzo et al. 1999). Differences may be due to fewer training days and no games reported for the soccer players, in addition to a lower body mass and FFM than the RL and RU players in this study. U20 players RMR was also ~ 750 Kcal day^− 1^ greater than junior basketball players (Silva et al. 2013). Despite observing a greater body mass, U20 players had a lower FFM than the basketball players. As previously mentioned TEE was greater in the basketball players, which reflects the more demanding training and game schedule. The greater RMR observed in this study may be due to increased body mass or due to the recovery cost from the collision aspects of rugby.

RL and RU U24 players RMR was ~ 350 Kcal day^− 1^ greater than senior RL players (Morehen et al. 2016). U24 players in this study weighed more and potentially had a similar FFM to RL U24 players (lean mass was reported), suggesting overall body mass may contribute towards increased energy demands rather than FFM alone. Some research suggests that greater lean mass increases RMR (Speakman and Selman 2003). In this study, U24 players had a greater body mass and FFM (12.3 and 12.3 kg) than U20 players, however, U20 players had a greater RMR when calculated relative to body mass or FFM. Consequently, FFM alone may not be a good predictor of RMR in rugby players.

It is more likely that the RMR for players in this study may have been elevated above their habitual RMR (i.e., no recovery cost), as it was not possible to restrict players from training 48 h prior to data collection. Given the structure of an in-season training period, where there is a decrease in intensity of external loads alongside greater match play when compared with pre-season (Black et al. 2017), it is unlikely a player will ever be in a completely rested state. RMR may increase by 5–10% for 36–48 h post-resistance training (Speakman and Selman 2003). Due to collisions, muscle damage is observed for 72–120 h post-match in rugby players (McLellan et al. 2011; Roe et al. 2016), which provides a further contribution to increased RMR. Therefore, the reported RMR in this study may be a more representative measure during the in-season period for practitioners to consider when providing nutrition or physiological support to athletes, although it is not a true rested physiological value.

A mean PAL of 2.0 ± 0.4 was observed for all players, which was lower than the 2.9 reported in senior RL players (Morehen et al. 2016). This may be due to a greater contribution of RMR (390–441 Kcal day^− 1^) to TEE in present study. Although a similar PAL for male adolescent athletes has been estimated as 2.0 ± 0.3 (Carlsohn et al. 2011), methods that predict BMR and TEE were used which may have resulted in under- or over-estimation of the PAL.

On an individual basis, metabolic rate prediction equations multiplied by an observed PAL resulted in an under-estimation of TEE by 3153 Kcal day^− 1^ (RU U24 Cunningham equation; Fig. 2) to an over-estimation by 1130 Kcal day^− 1^ (RU U16 Schofield equation; Fig. 2). Desbrow et al. (2014) suggest using the Schofield equation as a guide to estimate BMR in adolescents. Despite similar means between measured RMR and predicted BMR using the Schofield equation, there was a large degree of variability, and therefore unclear differences were observed for U16 players. ACSM (2016) suggest that the Cunningham and Harris–Benedict equations may provide a better estimate of RMR in athletes, than using alternative equations. However, the Cunningham equation under-estimated for U16 rugby players as a group (i.e., mean). All prediction equations under-estimated TEE for U20 players. Interestingly, when observing individual data, all prediction equations over-estimated for two players. PALs used in practise are often based on data by Black (2001), which was derived from mean data of 151 athletes, not including team sport players. Therefore, the PAL used in this study was specific to this rugby population and more accurate than using typical reference data.

In conclusion, there was a large variation between individuals for TEE and negligible mean differences observed between RL and RU players. RMR was greater for RL than RU players, greater for U20 than U24 players, and greater than other non-contact team sports, suggesting that differences in training exposure and match play, and the collision aspect of rugby may elevate RMR beyond an individual’s habitual state during an in-season period. Metabolic prediction equations multiplied by a PAL based on rugby specific empirical data typically under-estimated TEE. The TEE measured in this study using the gold standard DLW method can be used as reference data for elite rugby players of different codes and ages, during an in-season training period. The range of measured RMR and TEE highlights the importance of practitioners working with players on an individual basis when providing sport science support.

## Abbreviations

BMR: Basal metabolic rate
DLW: Doubly labelled water
DXA: Dual X-ray absorptiometry scans
FFM: Fat free mass
PAL: Physical activity level
RL: Rugby league
RMR: Resting metabolic rate
RU: Rugby union
TEE: Total energy expenditure
TEF: Thermic effect of feeding
